# Factors Influencing Job Retention and Quality of Life amongst Nasopharyngeal Carcinoma Patients

**DOI:** 10.31557/APJCP.2021.22.5.1401

**Published:** 2021-05

**Authors:** Narumon Janmunee, Thanarpan Peerawong, Tharin Phenwan, Sojirat Supanichwatana, Chanon Kongkamol

**Affiliations:** 1 *Department of Radiology, Faculty of Medicine, Prince of Songkla University, Hat Yai, Songkhla, Thailand. *; 2 *School of Medicine, Walailak University, Nakhon Si Thammarat, Thailand. *; 3 *School of Health Sciences, University of Dundee, DD14HJ, United Kingdom. *; 4 *Department of Education Foundation, Faculty of Liberal Arts, Prince of Songkla University Hat Yai, Songkhla, Thailand. *; 5 *Research Unit of Holistic Health and Safety Management in the Community, Faculty of Medicine, Prince of Songkla University, Hat Yai, Songkhla, Thailand. *

**Keywords:** Quality of life, nasopharyngeal carcinoma, job retention, FACT-NP

## Abstract

**Objective:**

To evaluate the quality of life (QoL) amongst Thai nasopharyngeal cancer patients (NCP) and identify associated factors with QoL.

**Methods:**

This study was based on secondary data from a cross-sectional study that aimed to develop the Thai version of functional assessment of cancer therapy with nasopharyngeal cancer subscale demographic data, clinical information of participants, and Functional Assessment of Cancer Therapy with Nasopharyngeal cancer subscale (FACT-NP) were utilized. Data were analyzed using Student’s t-test, rank-sum test, variance analysis, and the Kruskal-Wallis test. Multiple linear regression with the stepwise model was used to determine multiple variable analysis. Statistical significance was defined at p-value < 0.05.

**Results:**

Two hundred and thirty NCP were included in the study with a mean age of 50.3±12.4 years. According to our findings, 68.3% were male, 81.7% were married or living with a partner, and 86.1% were Buddhism had the Eastern Cooperative Oncology Group (ECOG) performance status between 0-2 (95.2 %). The employment status, education level, economic status, ECOG, stage , and disease status significantly influenced patients’ QoL. Patients who had active treatment and received prophylactic percutaneous gastrostomy were also impacted by the FACT-NP score. In the multivariate analysis, employment status, ECOG, and disease status were shown to be significant factors that were associated with their QOL in the final model.

**Conclusion:**

Employment status was a socioeconomic factor that led to positive QOL amongst NCP.

## Introduction

Nasopharyngeal carcinoma is less common in European countries and North America. However, it is more common in the eastern countries, such as China and Southeast Asia countries, including Thailand. The age-standardized rate (ASR) is 0.44 in European countries, 3.0 in China, and 2.2 in Thailand (Ferlay et al., 2019). Although this disease is included in the subsite of head and neck cancer, the treatment is different. Surgery, followed by radiation or chemoradiation, is a primary treatment choice in a non-metastatic head and neck cancer, while radical radiation therapy or concurrent chemoradiation is recommended in inoperable cancers in organ preservation cases. However, radiotherapy is the primary modality in non-metastatic nasopharyngeal carcinoma (Halperin et al., 2019). Concurrent chemoradiation, followed by adjuvant chemotherapy, is recommended in locally advanced diseases to improve progression-free and overall survival rates (Yan et al., 2015; Saba et al., 2016). However, treatment complications, such as chronic dysphagia, dry mouth, and hearing loss, often lead to additional suffering (Du et al., 2015).

Quality of life (QoL) is defined as “the individual’s perception of their position in life in the context of the culture and value systems in which they live and in relation to their goals.” (The WHOQOL Group, 1998). The patient’s well-being is one of the essential endpoints in cancer care. QoL is a multidimensional concept and usually involves subjective evaluations of both positive and negative aspects of life. In head and neck cancer, Functional Assessment of Cancer Therapy-Head and Neck (FACT-H&N) Scale and the European Organization for Research and Treatment of Cancer (EORTC) Quality of Life Head and Neck module (QLQ-H&N35) suggest measures to evaluate QoL in cancer patients (Ojo et al., 2012). These scales were developed for all types of head and neck cancers. Still, for nasopharyngeal carcinoma patients, Functional Assessment of Cancer Therapy with Nasopharyngeal cancer subscale (FACT-NP) was developed specifically for this disease due to differences in treatment toxicities profiles (Tong et al., 2009). 

Sociodemographic factors, such as age, gender, marital status, employment status, and education level can affect QoL in head and neck cancer patients. The disease status, such as tumor size and type, stage, and treatment type also affect the QoL in these patients (Bilal et al., 2015). Moreover, for nasopharyngeal carcinoma, radiotherapy techniques, such as intensity-modulated radiotherapy technique, influence patients’ QoL after treatment (Bian et al., 2015). However, no study has yet investigated QoL in Thai patients suffering from nasopharyngeal carcinoma. Especially in Southern Thailand, where Buddhism and Muslimism people are living together. This study aimed to evaluate nasopharyngeal carcinoma patients’ QoL and identify factors affecting their QoL.

## Materials and Methods

*Ethical consideration*

The study was approved by the Human Research and Ethics Committee of the Faculty of Medicine, Prince of Songkla University (REC: 56-006-07-1-3).

*Study design and setting*

This study was based on secondary data from a cross-sectional study that aimed to develop the Thai version of functional assessment of cancer therapy with nasopharyngeal cancer subscale (FACT-NP) (Peerawong et al., 2019). The study was conducted in the largest tertiary hospital in Southern Thailand. The radiation oncology unit treats approximately 100 new cases of nasopharyngeal carcinoma patients per year. The study was done between January 2014 and October 2016.

Inclusion criteria were patients with nasopharyngeal carcinoma (at any stage) who aged > 18 years. Patients who were diagnosed with other cancers, did not understand Thai, had delirium symptoms, or suffered from a major depressive disorder were excluded from the study.

*Instruments*

A researcher-made questionnaire comprising two parts was used for data collection. The first part included questions on patients’ sociodemographic information, disease stage, and treatment status, which was filled out by the research team. The second part was based on functional assessment of cancer therapy nasopharyngeal cancer (FACT-NP) subscale, which was translated and validated in Thai (Tong et al., 2009; Peerawong et al., 2019). The questionnaire involved five domains: physical well-being (PWB), social/family well-being (SWB), emotional well-being (EWB), functional well-being (FWB), and nasopharyngeal cancer subscales (NPS). The questionnaire comprised 43 items and a 5-point Likert scale (not at all to very much)was used for its scoring. The scores ranged from 0 to 64. The total score ranged from 0 to 172. Higher scores meant a higher QoL. 

*Statistical analysis*

Descriptive statistics were used to analyze patients’ sociodemographic information as well as, disease and treatment status. To compare two groups in terms of FACT-NP subscale scores, Student’s t-test was used. Rank-sum test was performed for non-normally distributed data. Spearman’s correlation was used for evaluating the correlation between age and FACT-NP subscale score. The analysis made comparisons of the different of the FACT-NP between at least three groups of variance analysis for normally distributed data, and the Kruskal-Wallis test for non-normally distributed data. Before multivariate analysis, the independent variables in which p-value < 0.2 were assessed multicollinearity with Variance Inflation Factors. Suppose the variance of inflation factors more than 10; those independent variables were excluded from models (Hair et al., 1995). The multiple linear regression with the stepwise model was used for determining multiple variables analysis. A p-value less than 0.05 was considered statistically significant. The reliability of translated FACT-NP subscale was determined using the Cronbach’s alpha. Statistical analysis was performed using the R Statistical Package.

**Table 1:** Sociodemographic and Clinical Characteristics of Participants (n=230)

Variables	n (%)
Age (mean and standard deviation)	50.3±12.4
Male	157 (68.3)
Religious	
Buddhism	198 (86.1)
Islamism	32 (13.9)
Status	
Single	28 (12.2)
Married or living with a partner	188 (81.7)
Divorce	14 (6.1)
Education level	
Bachelor and above	61 (26.5)
Secondary school	70 (30.4)
Primary school	92 (40.0)
Unlettered	7 (3.0)
Working	131 (57.0)
Economic problem	46 (20)
Stage	
I	9 (3.9)
II	21 (9.1)
III	90 (39.1)
IV	110 (47.8)
Eastern Cooperative Oncology Group performance status	
0 to 2	219 (95.2)
3 to 4	11 (4.8)
Active treatment	86 (37.4)
Disease status	
Loco-regional disease	73 (31.7)
No evidence of disease	129 (56.1)
Recurrence or metastases	28 (12.2)
Treatment	
Before treatment	67 (29.1)
Radiation	14 (6.1)
Chemo-radiation	147 (63.9)
Supportive treatment	2 (0.9)
Prophylactic gastrostomy	157 (68.3)

**Table 2:** Comparing QOL with Reference to Sociodemographic and Clinical Characteristics (n=230)

Factor (%)	QOL score (median (IQR), mean (SD))					
	PWB (0-28)	SWB (0-28)	EWB (0-24)	FWB (0-28)	NPS (0-64)	Total (0-172)
Employment						
Yes	26.6 (23.8,28.0)	22 (19.9,27.0)	24.0 (21.6,24.0)	21.0 (18.0,24.2)	49 (43.0,54.0)	142.6 (127.6,151.4)
No	23.8 (18.2,26.6)	21.0 (18.3,25.0)	21.6 (19.2,24.0)	19.0 (15.0,21.0)	42.0 (32.5,48.5)	125.2 (109.5,136.5)
p-value	< 0.001	0.179	< 0.001	< 0.001	< 0.001	< 0.001
Education						
University and above	26.6 (23.8,28.0)	24.0 (21.0,27.0)	24.0 (20.4,24.0)	21.5 (4.6)	49.0 (42.0,55.0)	143.6 (127.2,155.0)
Secondary school	25.2 (22.4,28.0)	21.0 (18.2,26.0)	22.8 (20.4,24.0)	19.8 (4.6)	46.0 (40.2,52.0)	133.4 (123.7,145.4)
Primary school and below	25.2 (20.3,26.6)	21.0 (18.0,26.4)	21.6 (19.2,24.0)	19.1 (4.5)	45.0 (36.0,49.0)	129.6 (115.6,141.5)
p-value	0.004	0.045	0.31	0.007	0.014	< 0.001
Economic problem						
Yes	25.2 (18.2,26.6)	22.0 (19.0,26.6)	21.6 (18.3,24.0)	17.5 (15.2,21.8)	43.0 (33.5,48.8)	129.5 (103.8,140.1)
No	25.2 (22.4,28.0)	21.0 (19.0,26.0)	22.8 (20.4,24.0)	20.0 (17.0,24.0)	46.5 (40.0,54.0)	135.4 (122.3,148.1)
p-value	0.018	0.967	0.005	0.018	0.003	0.003
ECOG						
0 to 2	25.2 (22.4,28.0)	21.0 (19.0,26.0)	22.8 (20.4,24.0)	20.0 (17.0,23.5)	46.0 (40.0,52.0)	135.0 (122.3,148.0)
3 to 4	16.8 (11.9,19.6)	23.3 (18.5,24.0)	20.4 (16.8,23.4)	14.0 (12.0,17.5)	24.0 (19.5,32.5)	99.0 (91.7,109.7)
p-value	< 0.001	0.54	0.115	< 0.001	< 0.001	< 0.001
Stage						
I	26.6 (25.2,28.0)	25 (21.0,26.0)	24.0 (21.6,24.0)	20.9 (6.8)	43 (42.0,49.0)	135 (127.2,143.2)
II	28 (25.2,28.0)	24 (21.0,28.0)	24 (20.4,24.0)	22 (4.5)	48 (44.0,51.0)	146.8 (135.2,151.2)
III	26.6 (22.4,28.0)	23.5 (21.0,27.0)	24 (20.4,24.0)	21.1 (4.4)	47 (40.2,53)	139.2 (126.0,148.9)
IV	24.5 (21,27.6)	21.0 (18.0,25.0)	21.6 (19.5,24)	18.6 (4.3)	44.0 (35.5,50.0)	126.7 (113.2,141.8)
p-value	0.011	0.015	0.195	< 0.001	0.114	< 0.001
Disease status						
Loco-regional disease	23.8 (19.6,26.6)	21.0 (18.0,24.0)	21.6 (19.2,24.0)	18 (4.2)	45.0 (40.0,50.0)	125.6 (115.4,140.0)
No evidence of disease	26.6 (25.2,28)	24.0 (20.0,28.0)	24.0 (21.6,24.0)	21.6 (4.2)	47.0 (41.0,54.0)	142 (128.6,151.8)
Recurrence or metastases	22.4 (18.9,25.5)	21.0 (18.0,26.0)	21.6 (17.7,22.8)	17.5 (4.6)	37.0 (23.8,46.0)	120.6 (95.3,131.0)
p-value	< 0.001	0.002	< 0.001	< 0.001	< 0.001	< 0.001
Active treatment						
Yes	23.8 (19.6,26.6)	21.0 (18.0,24.8)	21.6 (19.2,24.0)	18.0 (15.0,20.0)	44.0 (37.2,50)	124.9 (113.8,137.5)
No	26.6 (23.8,28)	23.5 (20.0,28.0)	24.0 (20.4,24.0)	21.0 (18.0,25.0)	47.0 (39.8,53.2)	139.9 (126.0,151.0)
p-value	< 0.001	< 0.001	< 0.001	< 0.001	0.12	< 0.001
Prophylactic gastrostomy						
Yes	25.2 (22.4,28.0)	22.0 (20.0,27.0)	24.0 (20.4,24.0)	21.0 (17.0,24.0)	46.0 (39.0,52.0)	128.6 (113.4,143.2)
No	25.2 (19.6,28.0)	21.0 (18.0,25.0)	21.6 (19.2,24.0)	19.0 (15.0,22.0)	46.0 (37.0,51.0)	136.6 (122.8,148.0)
p-value	0.097	0.016	0.039	0.01	0.628	0.027

QOL, quality of life; IQR, interquartile ranges; SD, standard deviation; PWB, physical well-being; SWB, family/social well-being; EWB, emotional well-being; FWB, functional well-being.The statistic calculated by Rank sum test; t-test; Kruskal-Wallis test; ANOVA

**Table 3:** Multivariate Linear Regression Analysis of Sociodemographic and Clinical Characteristics

Variables		Coeff	95.0% CI	p-value
Intercept		124.4	119.61 -129.17	<0.001
Employment status				
Reference	No			
	yes	10.58	5.68- 15.48	<0.001
ECOG				
Reference	0 to 2			
	3 to 4	-23.86	-22.3	<0.001
Disease status				
Reference	Loco-regional disease			
	No evidence of disease	7.06	1.80 -12.38	<0.001
	Recurrence or metastases	-12.27	-15.33	<0.001

CI, confident interval

## Results

*Participant characteristics*

Two hundred and thirty patients who completed the questionnaire were included in the study. According to our findings, the patients’ mean age was 50.3 (±12.4) years, 68.3% were male , 81.7% were married or living with a partner, and 86.1% were Buddhism. Fifty-seven percent had secondary school education, and fifty-seven percent of them were employed. Nevertheless, 20% of them had an economic problem. Eighty-eight percent of the patients were diagnosed with stage III or IV nasopharyngeal carcinoma. Almost all of them were ambulatory and capable of self-care, but they could not carry out work-related activities more than half of their waking hours (ECOG 2). Thirty-seven percent of them were still receiving treatment, while 56.1% had no evidence of disease (see Table 1). 

*QoL scores and the associated factors*

The reliability of the second part of the questionnaire was 0.92. The median (minimum and maximum) of FACT-NP subscale was 129.8 (58,168). The score of each domain was 25.2 (5.6,28), 21.5 (5,28), 20 (7,28), 22.8 (4.8,24), and 46 (13,62) for PWB, SWB, EWB, FWB, and NPS, respectively. 

Through univariate analysis, the relation between patients’ QoL and sociodemographic characteristics, disease stage and status, and treatment status were determined. The variables of employment status, education level, financial status, ECOG , disease stage, disease status, receiving active treatment, and performing prophylactic gastrostomy were significantly associated with patients’ QoL (Table 2). Disease status had a significant influence on all domains of the FACT-NP subscale. All variables affected PWB domain except for receiving PEG, which was not significant. 

Regarding SWB domain, education level, cancer stage, disease status, receiving active treatment, and performing PEG influenced the patients’ QoL score. The education level, ECOG status, and stage of cancer had no significant influence on EWB. All significant variables had an impact on FWB. 

With respect to NPS domain, all sociodemographic variables influenced the patients’ QoL. At the same time, ECOG status and disease status influenced the NPS domain.

According to the multiple linear regression analysis using the stepwise model, being employed had a significant effect on the patients’ QoL. While patients who were not ambulatory, capable of self-care but unable to carry out any work-related activities for more than half of their waking hours (ECOG 3-4 ) negatively impacted the QOL score. Comparison with patients with a loco-regional disease, patients who had no evidence of disease had higher QoL scores. While patients with recurrence or metastases had significantly lower scores (see Table 3). 

## Discussion

This study aimed to determine factors associated with QoL in patients with nasopharyngeal carcinoma. The results showed that employment status, ECOG, and disease status influenced nasopharyngeal carcinoma patients’ QoL. From this study, only Employment status was the single sociodemographic that was changeable, whereas ECOG and disease status were parts of clinical determination and treatments. 

Treatments for head and neck cancer can cause a change in the voice, loss of hearing, trouble swallowing, and hair loss (Halperin et al., 2019). These changes have a drastic impact on patients’ daily life function, their body image, and socialization capability (Miller, 2020). The treatment of nasopharyngeal carcinoma includes seven to eight weeks of concurrent chemoradiation plus twelve weeks of adjuvant three cycles of chemotherapy (Lee et al., 2009; Peerawong et al., 2012). The treatment can last up to five months. Due to prolonged treatment duration, patients have to change their daily life and work activities or need sick leave to continue their treatment. However, in an agricultural-bound society such as Southern Thailand, patients have to quit their job. Bilal et al., (2015) conducted a cross-sectional study in Malaysia, which included 361 head and neck cancer patients. They found that employment status was associated with patients’ QoL score (Bilal et al., 2015), which is in line with our findings. Return to work (RTW) is an important issue that is overlooked in cancer care (Lee et al., 2015). The RTW rate in head and neck cancer patients worldwide varies from 32% to 92% (Miller, 2020). In the developing countries, a prospective study from India revealed an 85% RTW rate within 19 months after the treatment (Agarwal et al., 2017). In this study, 24.4% of the participants have received chemoradiation. Participants who received chemoradiation had a RTW rate of 25.7%. In comparison, participants who received surgery and radiotherapy had a RTW rate of 41.9% (Agarwal et al., 2017). It seems that the treatment type may influence RTW, too. In our study, only 57% of the patients were still going to work. About 20% of the patients also reported financial difficulties after their treatment. Our results revealed that employment rate was higher than that in head and neck cancer patients in Malaysia (Bilal et al., 2015). 

The employment status affected our patients’ PWB, EWB, FWB, and NPS, but it did not affect their SWB that can be due to this fact that Thai society is a collectivist society. Then, unemployment did not impact this aspect. The physical changes after treatment, such as stiff neck, taste change, and dry mouth influenced physical functioning and daily life activities of our patients. and affected to emotional aspect by unacceptable self-transformation. People were harmony of the whole family unit could affect their quality of life (Hofstede et al., 2010).

A previous study on spiritual well-being of Thai patients with breast cancer revealed that family was one of the significant factors that affected patients’ spiritual well-being (Phenwan et al., 2019). Thus, as long as the family’s harmony is retained, patients’ SWB will be intact. However, Phenwan’s study (2019 ) on breast cancer survivors revealed the importance of SWB amongst Southern Thais. This discrepancy can be due to this fact that this area has pluralistic culture. Thai Buddhists live in harmony with Thai Muslims, creating a unique harmony in the area. Still, the spirituality of Southern Thai people with head and neck cancer can be explored further in the future. 

People with nasopharyngeal carcinoma have long term survival. A meta-analysis results showed that the 5-year and 10-year overall survival rate was 62.2% and 53.2%, respectively (Blanchard et al., 2015). Then they had a transition period in their life since from cancer diagnosed to normalization. A previous study on Thai breast cancer survivors revealed that there were three phases of transformation: (1) the moment of diagnosis and changed self, (2) transition and recovery, and (3) normalization. In each phase, people with cancer needed additional support such as in the first phase; they need good truth-telling and respect their autonomy (Peerawong et al., 2019).

It can be concluded that employment status and return to work are a part of body image transformation in survivors’ life. In the normalization phase, patients need family and social support and social construction. Then future research in return to work of the survivors, Patient public involvement should be a concern. 

To our knowledge, our work was the first study exploring employment status as one of the factors affecting QoL in NCP patients. In the individualized medicine era, the intensity-modulated radiotherapy technique (IMRT) can reduce radiation toxicities and improve patients’ QoL (Bian et al., 2015). The additional patient’s voice, such as their occupation in radiation treatment design, additional to other clinical factors, may genuinely be individualized. 

Our study also had limitations. First, this study was conducted in a single health care center. IMRT did not do for all patients. The QoL score may be underestimated. Future multicenter prospective study is suggested to be conducted to identify RTW rate and QoL associated factors. Second, our study did not discern the importance of employment status and the RTW process in patients with nasopharyngeal carcinoma. 

In conclusion, job retention is an independent factor that influenced nasopharyngeal carcinoma patients’ QoL. For better understanding and improving these patients’ QoL, future studies should be conducted.

## Author Contribution Statement

Thanarpan Peerawong and Chanon Kongkamol conceived, designed, and performed the research. Sojirat Supanichwatana analyzed the data. Narumon Janmunee, Thanarpan Peerawong and Tharin Phenwan wrote the manuscript. Tharin Phenwan edited manuscript.
